# The Role of Nailfold Videocapillaroscopy (NVC) in Evaluating Ocular Diseases: Insights into Retinal, Choroidal, and Optic Nerve Pathologies

**DOI:** 10.3390/jcm15030931

**Published:** 2026-01-23

**Authors:** Małgorzata Latalska, Magdalena Wójciak, Agnieszka Skalska-Kamińska, Sławomir Dresler

**Affiliations:** 1 Department of General and Pediatric Ophthalmology, Medical University of Lublin, 20-079 Lublin, Poland; 2 Department of Analytical Chemistry, Medical University of Lublin, Chodźki 4a, 20-093 Lublin, Poland; magdalena.wojciak@umlub.edu.pl (M.W.); agnieszka.skalska-kaminska@umlub.pl (A.S.-K.); slawomir.dresler@umlub.edu.pl (S.D.); 3 Department of Plant Physiology and Biophysics, Institute of Biological Sciences, Faculty of Biology and Biotechnology, Maria Curie-Skłodowska University, 19 Akademicka St., 20-033 Lublin, Poland

**Keywords:** microcirculation dysfunction, nailfold videocapillaroscopy, glaucoma, central serous chorioretinopathy, retinopathy

## Abstract

**Background/Objectives**: Nailfold videocapillaroscopy (NVC) is a non-invasive method for visualizing systemic micro-circulation, primarily used in rheumatology. Many ocular diseases (e.g., glaucoma, diabetic retinopathy (DR), and central serous chorioretinopathy (CSC)) involve microvascular disturbances. Since microangiopathies are often systemic, NVC findings may reflect ocular pathology. This narrative review aimed to summarize current evidence linking NVC alterations with retinal, choroidal, and optic nerve diseases. **Methods**: A literature search of PubMed, Scopus, and Web of Science (2000–2025) was conducted using the keywords “nailfold videocapillaroscopy,” “ocular diseases,” “retinopathy,” and “glaucoma”. **Results**: Most available studies were cross-sectional and exploratory. In glaucoma, NVC abnormalities suggesting systemic hypoperfusion (reduced capillary density, avascular areas, tortuosity, and microhemorrhages) were frequently reported. CSC was associated with capillary dilation patterns (megacapillaries and aneurysmal dilations), supporting a congestive rather than ischemic microvascular profile. In DR, NVC abnormalities (reduced density and neoangiogenesis) correlated with DR severity. Associations were also found for AMD and idiopathic macular telangiectasia type 2 (MacTel2, also known as IMT). However, only a limited number of prospective studies assessed diagnostic performance, and data on sensitivity, specificity, or ROC-based validation remain scarce. **Conclusions**: Current evidence suggests that NVC reflects systemic microvascular alterations associated with several ocular diseases. While NVC shows potential as an adjunctive tool for risk assessment and phenotyping, its diagnostic validity has not yet been established. Limitations include the predominantly observational nature of the studies, heterogeneity of methodologies, and the lack of standardized diagnostic thresholds. Prospective trials integrating NVC with ocular imaging modalities, such as OCT angiography, are needed to determine its clinical utility.

## 1. Introduction

**Figure 1:**
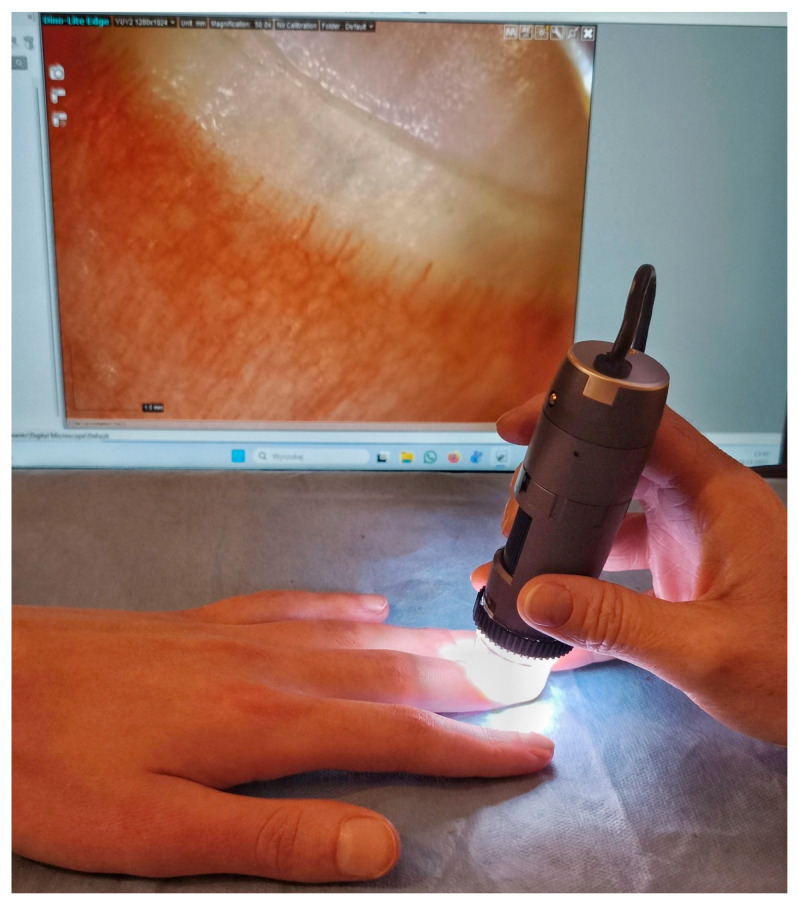
Representative image of nailfold videocapillaroscopy (NVC). Images were obtained using a Dino-Lite digital microscope (AnMo Electronics Corp., New Taipei City, Taiwan).

Nailfold videocapillaroscopy (NVC) is a non-invasive diagnostic method that enables direct observation of the microcirculation by capturing detailed images of capillary loops within the nailfold [1]. This region represents the terminal segment of the systemic vascular network, making it an ideal site for evaluating microvascular health. The examination is performed with a stereomicroscope equipped with a high-resolution digital camera, which allows real-time visualization of the capillaries responsible for tissue perfusion (Figure 1).

In this area, the capillaries run parallel to the skin surface, providing an optimal view of their shape, density, and structural abnormalities. While conventional nailfold capillaroscopy (NFC) involves simple visual inspection of the nailfold using a dermatoscope or light microscope and provides mainly qualitative information, NVC employs digital video microscopy with high magnification and computer-assisted image analysis, enabling quantitative and reproducible assessment of capillary morphology and density. The microcirculation itself consists of arterioles, venules, and capillaries—the smallest vessels, typically less than 20 µm in diameter—the function of which is to facilitate the exchange of oxygen and nutrients between blood and tissues. The origins of capillaroscopic observation date back to the 19th century, when the Italian physician Giovanni Rasori (1766–1873) first used a magnifying glass to examine nailfold capillaries, laying the foundation for modern microcirculatory assessment.

Because the nailfold microvasculature reflects systemic vascular integrity, NVC serves as a valuable window into generalized microcirculatory function [2]. Primarily, NVC has been an essential tool in rheumatology, particularly for diagnosing and monitoring connective tissue diseases such as systemic sclerosis and systemic lupus erythematosus, as well as for the assessment of Raynaud’s phenomenon [3]. In recent years, however, its use has expanded to include the assessment of microvascular involvement in conditions such as chronic kidney disease, diabetes mellitus, cardiovascular disorders, and dermatological diseases [4,5,6]. The evidence also suggests that NVC may offer valuable insights into ophthalmology, where systemic microvascular alterations can reflect or even precede changes observed in retinal, choroidal, and optic nerve circulation.

The eye possesses a highly complex and sensitive microvascular network. Proper capillary blood flow within the retina, choroid, and optic nerve head is essential for maintaining normal visual function. Microcirculatory disturbances play a significant role in the pathogenesis of various ocular disorders, such as diabetic retinopathy, glaucoma, and age-related macular degeneration (AMD). Since microangiopathies often have a systemic nature, alterations in nailfold capillary morphology may reflect similar changes occurring in the ocular microvasculature.

### Nailfold Videocapillaroscopy (NVC) vs. Nailfold Capillaroscopy (NFC)

Nailfold capillaroscopy (NFC), a traditional method for assessing microcirculation by direct visualization of nailfold capillaries, typically employs a dermatoscope or a simple microscope. However, this approach provides only qualitative information and is limited by the subjectivity of the observer. In contrast, nailfold videocapillaroscopy (NVC) utilizes a high-resolution digital microscope with magnification of up to ×600, enabling precise and quantitative evaluation of capillary morphology, density, and architecture.

The primary advantage of NVC is its ability to record video sequences. This capability allows for the assessment of dynamic features of blood flow and the extraction of high-quality frames for offline processing. This significantly increases diagnostic accuracy compared to conventional methods such as NFC.

There is some inconsistency in the terminology used in the literature regarding nailfold examination techniques. For example, Goh et al. described their method as nailfold capillaroscopy (NFC); however, they employed a digital video microscope with ×500 magnification and computer-assisted image capture and analysis, which corresponds more accurately to nailfold videocapillaroscopy (NVC) [7]. Similarly, Shikama et al. referred to their technique as nailfold capillaroscopy [8]. However, they used a digital microscope (approximately ×390 magnification) with image transmission to a computer and digital storage software, clearly indicating the use of a videocapillaroscopic system [8]. The same applies to Küçük et al., who described their procedure as NFC but used a digital Videocap system with ×200 magnification and computerized image recording, which should be classified as NVC [9]. Such inconsistencies in nomenclature may hinder the comparison of findings across studies and underscore the need for standardized terminology that distinguishes conventional NFC from digital NVC.

NVC ensures greater reproducibility and diagnostic accuracy, making it a preferred tool in modern research on systemic and ocular microvascular disorders. The comparison of NVC and NFC is summarized in Table 1.

Furthermore, the development of digital health technologies facilitates automated image analysis. Since both NVC and OCT-A, commonly used in ophthalmology, provide high-resolution quantitative data, they appear to be excellent tools for Artificial Intelligence applications. As noted in recent meta-analyses on ‘Explainable AI in Clinical Decision Support Systems,’ AI has the potential to objectify the assessment of such microvascular patterns and minimize observer bias [10]. However, successful implementation requires longitudinal clinical validation and uniform interpretability measures. Furthermore, one must consider the anatomical differences between vascular beds to establish consistent standards for interpretation.

The aim of this review is to discuss the role of nailfold videocapillaroscopy (NVC) in the evaluation of ocular diseases and to summarize current evidence on the associations between nailfold microvascular changes and retinal, choroidal, and optic nerve pathologies. This article seeks to highlight the potential diagnostic value of NVC in ophthalmology and to identify future directions for research exploring its clinical applicability in eye disease assessment.

## 2. Search Strategy

A comprehensive literature search was conducted to identify studies evaluating the role of nailfold videocapillaroscopy (NVC) in ocular diseases. The search was performed across several electronic databases, including PubMed, Scopus, and Web of Science, covering articles from 2000 to 2025. The following keywords and terms were used in various combinations: “nailfold videocapillaroscopy”, “capillaroscopy”, “microcirculation”, “ocular diseases”, “retina”, “choroid”, “optic nerve”, “retinopathy”, “glaucoma”, “retinal vein occlusion” and “uveitis”.

**Figure 2:**
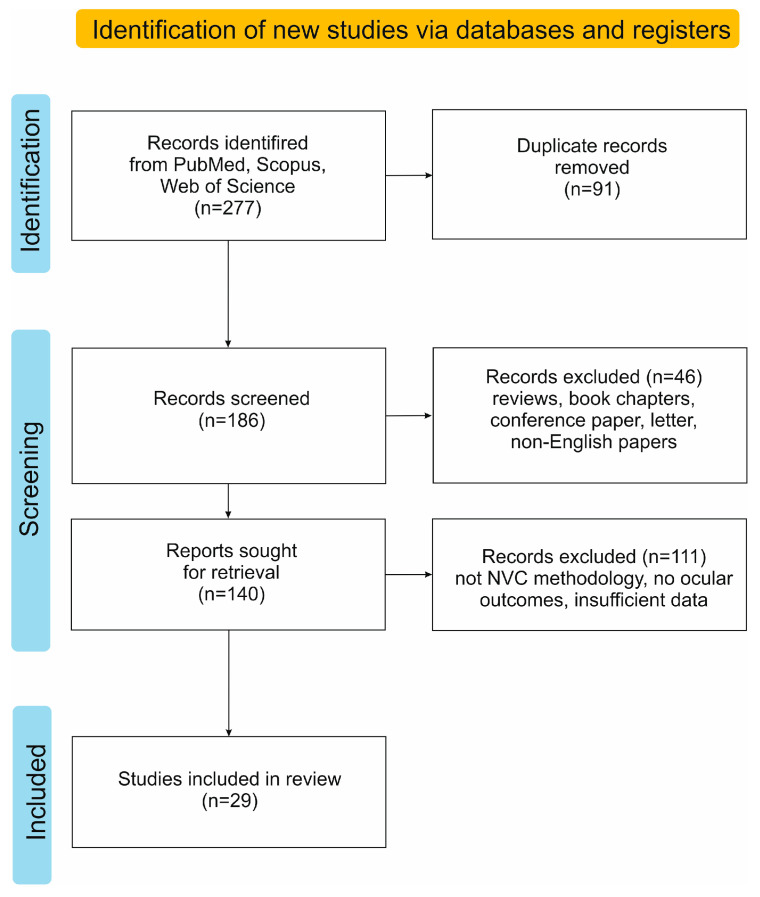
PRISMA flow diagram of the study selection process.

Boolean operators (“AND”, “OR”) were applied to refine and expand the search where appropriate. Only original research articles and case reports written in English were included. Reference lists of relevant articles were manually screened for additional eligible studies. Studies focusing on non-ocular diseases or employing methods other than NVC for microvascular assessment were excluded. PRISMA flow diagram of the study selection process is presented in Figure 2.

To ensure methodological consistency and address the heterogeneity of the included conditions (e.g., glaucoma vs. macular diseases), a rigorous operationalization of NVC parameters was applied during data extraction. Each study was evaluated not only for qualitative morphological signs but also for quantitative metrics, such as capillary density, loop diameter (apical, arterial, and venous limbs), and blood flow velocity (Vrest and Vmax). To mitigate the confounding effect of biological aging, we performed a systematic audit of control groups. Studies were prioritized based on the quality of their age-matching protocols (*p*-values for age ranging from 0.111 to 0.901) or on the use of advanced statistical adjustments (e.g., GEE—Generalized Estimating Equations, Cox Proportional Hazards Models). Furthermore, the methodological quality of each included study was objectively assessed using the Newcastle–Ottawa Scale (NOS), with scores incorporated into the comparative analysis to ensure the reliability of the synthesized evidence. This approach ensures that the observed microvascular alterations are independent of physiological aging and directly reflect the pathophysiology of the specific ocular condition.

## 3. Pathophysiology of Ocular Microvascular Changes

Understanding the systemic nature of microvascular dysfunction is crucial. It is the foundation that may explain the changes observed simultaneously in the distant nailfold microcirculation (NVC) and in the choroid and retina of the eye. However, for such a comparison to have scientific and clinical value, it is not enough to state generally that dysfunction exists in both locations. It is crucial to understand whether the specific morphological features observed in NVC (e.g., capillary rarefaction, megacapillaries, hemorrhages) correspond to specific pathophysiological patterns in the eye.

The specific pathways leading to ocular pathology vary; different primary insults result in unique diseases. This section describes the three main types of microvascular damage observed in the eye: (1) ischemia and rarefaction, (2) congestion and dysregulation, and (3) hypoperfusion and instability.

**Figure 3:**
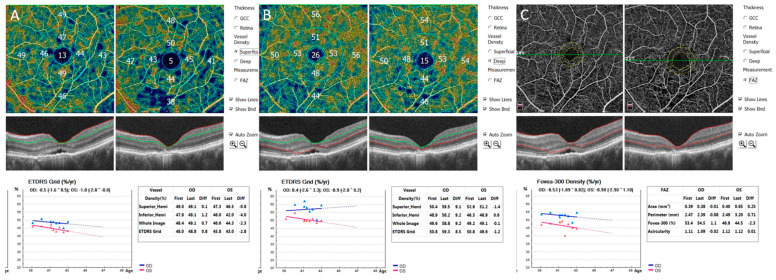
Optical Coherence Tomography Angiography (OCT-A) examination showing longitudinal follow-up. The top row displays en face vascular maps, the middle row shows corresponding cross-sectional OCT B-scan, and the bottom row presents quantitative data and progression graphs: ETDRS (Early Treatment Diabetic Retinopathy Study Grid). (**A**) Vessel Density analysis of the Superficial Capillary Plexus (SCP); (**B**) Vessel Density analysis of Deep Capillary Plexus (DCP); (**C**) Measurement and analysis of the Foveal Avascular Zone (FAZ). Images were acquired using Angio Retina QuickVue, Angio Retina, and Cross Line scans (Algorithm Version A2017.1.0.151; Optovue, Inc., Fremont, CA, USA).

Advances in modern imaging techniques, particularly optical coherence tomography angiography (OCT-A), have significantly improved our ability to differentiate these processes in vivo in the eye. OCT-A enables the quantitative analysis of retinal vessel density (RVD) and the foveal avascular zone (FAZ) (Figure 3).

Therefore, the fundamental question is whether, for example, an “ischemic” pattern in NVC (capillary loss) correlates with an “ischemic” pattern in the retina (a drop in VD on OCT-A), and whether a “congestive” pattern (e.g., megacapillaries) correlates with changes in the choroid. Understanding these analogies is key to validating NVC as a systemic biomarker for eye diseases.

### 3.1. Ischemia and Rarefaction: Diabetic and Autoimmune Microangiopathy

In diseases such as diabetes mellitus and systemic autoimmune conditions (e.g., systemic sclerosis, SSc), the final microvascular process leads to capillary rarefaction. However, the underlying pathways differ significantly. In diabetic retinopathy (DR), the pathology includes pericyte loss and basement membrane thickening, both consequences of hyperglycemia. In contrast, SSc is characterized by autoimmune-mediated endothelial cell injury followed by fibrosis. Nevertheless, despite these distinct mechanisms, both conditions ultimately result in capillary nonperfusion and tissue ischemia.

In the eye, this process manifests as retinal ischemia. In diabetic retinopathy (DR), OCT-A reveals this progression through quantifiable metrics: reduced vessel density in the superficial and deep capillary plexuses, enlargement of the foveal avascular zone (FAZ), and the appearance of “capillary dropout” areas. This process also involves choroidal thinning and chronic ischemia triggers compensatory mechanisms, such as the upregulation of vascular endothelial growth factor (VEGF), leading to neovascularization [11].

Similarly, the systemic vasculopathy of SSc also affects the eye. The literature confirms that SSc is associated with posterior segment pathologies, including retinal microcirculatory issues and optic nerve abnormalities [12]. The underlying mechanism is consistent with the systemic disease: a progressive loss of capillaries (rarefaction) leading to chronic tissue hypoperfusion and ischemia, a process that can be correlated with peripheral NVC findings [13].

### 3.2. Congestion and Dysregulation: The Pachychoroid Spectrum

A contrasting pathophysiological mechanism underlies pachychoroid spectrum diseases, which include central serous chorioretinopathy (CSC). The primary feature here is not capillary loss, but vascular congestion and dysregulation. The term “pachychoroid” refers to a pathological increase in choroidal thickness, caused by the dilation of outer choroidal vessels (known as “pachyvessels”). This dilation is hypothesized to result from impaired venous outflow or generalized vascular dysregulation. This chronic congestion leads to the mechanical compression of the overlying, more fragile choriocapillaris layer. The compression and subsequent ischemia of the choriocapillaris are believed to impair the function of the retinal pigment epithelium (RPE), leading to RPE dysfunction, fluid leakage, and the characteristic serous retinal detachments seen in CSC [14].

### 3.3. Hypoperfusion and Instability: Glaucomatous Optic Neuropathy

The mechanical stress on the lamina cribrosa is a well-established cause of damage in high-tension glaucoma, such as primary open-angle glaucoma (POAG). However, this pressure-centric model remains insufficient for explaining Normal-Tension Glaucoma (NTG), in which intraocular pressure remains within physiological limits, and the primary source of damage is vascular rather than mechanical. Consequently, the primary mechanism shifts from physical compression to vascular dysregulation and hypoperfusion of the optic nerve head (ONH), which is distinct from the chronic ischemia typical of diabetic retinopathy [15,16].

OCT-A studies consistently demonstrate reduced vessel density in the radial peripapillary capillary plexus (RPC) and within the ONH itself. This localized hypoperfusion is considered a primary driver of neurodegeneration. This dysregulation may manifest as an inability to autoregulate blood flow in response to changes in intraocular or systemic pressure, or as a systemic tendency toward vasospasm, leading to intermittent and unstable perfusion, which is particularly damaging to the metabolically active axons of the optic nerve [17,18].

### 3.4. Anatomical and Physiological Distinctions

While the findings summarized here suggest a parallel between nailfold and ocular microvascular changes, the physiological differences between these two vascular beds must be emphasized. The retinal circulation is protected by the blood–retina barrier (BRB) and exhibits robust local autoregulation, whereas the nailfold microvasculature lacks such a barrier and is highly sensitive to autonomic and thermal stimuli. Therefore, NVC findings should not be interpreted as a direct mirror of retinal hemodynamics. Instead, OCT-A and NVC should be viewed as complementary imaging modalities: OCT-A provides high-resolution data on the ‘end-vascular bed’ (the eye) damage, while NVC serves as a surrogate marker of the underlying systemic endothelial status. The co-occurrence of abnormalities in both beds—despite their distinct physiological regulations—strengthens the hypothesis that certain ocular diseases are manifestations of a generalized, systemic microvascular vulnerability.

## 4. Nailfold Videocapillaroscopy Findings in Ocular Disorders

### 4.1. NVC Parameters Relevant to Ocular Diseases

Among the various parameters assessed by nailfold videocapillaroscopy, several have particular relevance for understanding microvascular involvement in ocular disorders [19]. Capillary density is one of the most informative indicators, as its reduction reflects systemic microvascular rarefaction and impaired tissue perfusion [20]. Decreased capillary density observed in NVC has been correlated with reduced ocular blood flow, particularly in conditions such as normal-tension glaucoma and systemic sclerosis, where optic nerve hypoperfusion plays a key pathogenic role [7,15]. Capillary morphology also provides valuable information. Structural irregularities, including tortuous, dilated, or bushy capillaries, may indicate vascular dysregulation or endothelial dysfunction, mechanisms that are likewise implicated in retinal and choroidal microangiopathies. The presence of giant or dilated capillaries suggests compensatory vasodilation secondary to hypoxia, analogous to the vascular remodeling observed in choroidal ischemia or early diabetic retinopathy.

Another relevant feature is the occurrence of microhemorrhages, which point to increased vascular fragility and local endothelial damage. Such findings may parallel hemorrhagic manifestations in ocular microvasculature, for example, in retinal vein occlusion or diabetic microangiopathy. Likewise, the identification of avascular areas—regions where capillary loops are absent—signals localized ischemia and impaired perfusion, potentially mirroring reduced retinal or choroidal blood flow. Finally, evidence of neoangiogenesis in NVC, though less frequent, may reflect chronic tissue hypoxia and angiogenic activation, mechanisms also characteristic of proliferative retinopathies. Altogether, these NVC parameters provide a noninvasive window into systemic microvascular health and may serve as peripheral correlates of ocular vascular pathology.

It should also be noted that capillary microscopy patterns are influenced by age, and this factor should be taken into account when interpreting NVC findings. Piette et al., in a study including 100 patients aged over 65 years and 100 young healthy adults, found a higher prevalence of arteriovenous sludge, increased capillary loop length, and a more prominent subpapillary plexus in older patients [21]. Therefore, these capillary patterns should be regarded as age-related rather than pathological in individuals over 65 years of age. Table 2 provides a conceptual framework for the parallel morphological changes in systemic and ocular microcirculation, while the quantitative data and statistical rigor of specific studies are detailed in Table 3.

### 4.2. Nailfold Videocapillaroscopy (NVC) in Various Ocular Diseases

Nailfold videocapillaroscopy (NVC) has emerged as a valuable, non-invasive method for visualizing peripheral microcirculation. In recent years, growing evidence has highlighted the close interplay between ocular and systemic vascular conditions [20]. Because many ophthalmic diseases are linked to microvascular dysregulation, NVC has become an increasingly useful adjunctive tool in ophthalmology for exploring systemic vascular alterations that may accompany or contribute to ocular pathology.

In ophthalmology, this technique has most commonly been used to evaluate microcirculatory parameters in various types of glaucoma. For example, Cousins et al. observed that patients with primary open-angle glaucoma (POAG) as well as those with exfoliation syndrome with or without glaucoma (XFS/XFG) exhibited greater nailfold hemorrhages, avascular zones, and capillary tortuosity compared to controls, indicating systemic microvascular impairment [22]. In another study, Cousin et al. have measured dynamic blood flow in POAG patients. Using linear mixed models to adjust for age, gender, and mean arterial pressure, they identified a reduction in resting capillary blood flow velocity in POAG patients (26.8 ± 17.6 pL/s vs. 50.1 ± 24.2 pL/s; <0.0001) independent of covariates such as blood pressure, pulse and IOP [18]. They also found that the number of hemorrhages per 100 capillaries was comparable between XFS/XFG and POAG patients; however, the XFS/XFG group exhibited a greater number of avascular zones and more pronounced tortuosity [22]. Pasquale et al. confirmed these hemorrhages, dilated loops, and avascular zones in healthy subjects independent of disease severity [15]. Kosior-Jarecka et al. further demonstrated that NTG patients present characteristic NVC abnormalities—microbleedings, branching, and enlarged capillaries—correlating with markers of disease severity, including higher intraocular pressure and optic disc hemorrhages [16]. Łukasik et al. applied NVC to evaluate microvascular alterations in patients with pseudoexfoliative glaucoma (XFG) and to explore potential associations between NVC patterns and hypertensive (hXFG) or normotensive (nXFG) subtypes. Although the overall frequency of abnormal NVC patterns did not differ significantly between XFG patients and controls, several distinct microvascular abnormalities were identified. Microhemorrhages were more frequently observed in the nXFG group compared with controls (30.0% vs. 6.25%), and a higher prevalence of capillary tortuosity was noted in XFG patients, particularly in those with advanced glaucomatous neuropathy. Moreover, capillary dilatation and microbleedings were most often detected in patients with lower intraocular pressure values. Notably, tortuosity was significantly more common in pseudoexfoliative glaucoma than in healthy individuals [23].

Recent research has concentrated more on quantitative analysis than qualitative morphological descriptions. Shoji et al. used Generalized Estimating Equations (GEE) to control for confounders, including age, sex, and mean arterial pressure (MAP) [24]. They found a statistically significant reduction in mean capillary diameter in glaucoma patients compared to matched controls (14.5 ± 2.4 µm vs. 15.7 ± 1.6 µm; *p* = 0.036), and a significant decrease in capillary length (*p* = 0.004). Furthermore, quantitative measurements of peripheral blood flow velocity provide fascinating physiological context. Recent studies demonstrated that glaucoma patients exhibit significantly lower resting blood flow velocity and reduced peak flow during post-occlusive reactive hyperemia compared to healthy individuals [18,25]. These findings appear to provide objective biomarkers linking systemic microvascular characteristics to optic nerve pathologies.

Only one study has reported no association between NVC findings and glaucoma. Gomes et al. found no statistically significant relationship between glaucoma diagnosis and NVC patterns in patients with systemic sclerosis (SSc) [26]. In this study, the control group consisted of SSc patients without glaucoma, which may explain the lack of difference. Jakhar et al. also did not notice differences in NFC features in SSc patients with and without retinal changes [27]. Since SSc itself is characterized by pronounced microvascular abnormalities, these systemic changes could have masked any additional microcirculatory alterations associated with glaucoma [28,29]. This finding suggests that, in patients with pre-existing systemic vasculopathy such as SSc, NVC may have limited sensitivity in distinguishing ocular-specific microvascular dysfunction.

Apart from glaucoma, the usefulness of NVC has also been verified in another common ophthalmic condition, namely central serous chorioretinopathy (CSC). Erol et al. applied NVC in patients with CSC, revealing clear evidence of systemic microvascular alterations. CSC patients showed reduced capillary density and increased frequencies of abnormalities such as capillary ectasia, aneurysmal dilatation, microhemorrhages, avascular areas, and tortuosity, suggesting generalized endothelial dysfunction [30]. These findings were expanded by Latalska et al., who demonstrated that abnormal NVC patterns appeared only in CSC patients, characterized by specific microvascular changes including dilated apical parts of capillaries, microaneurysmal dilations, megacapillaries, fresh microhemorrhages, and bizarre or broken capillary loops [31]. In a subsequent study, the same authors examined various CSC subtypes and confirmed the exclusive presence of abnormal NVC findings in these patients, with distinct vascular profiles depending on disease form. Meandering capillaries predominated in acute and recurrent CSC, while glomerular and aneurysmal dilations were more characteristic of chronic and neovascular types [32].

The usefulness of NVC has also been confirmed in patients with diabetic retinopathy (DR). Bakirci et al. demonstrated that patients with DR exhibit characteristic NVC abnormalities, including reduced capillary density, increased capillary tortuosity, and the presence of microhemorrhages, compared with diabetic patients without retinopathy (DM) [33]. In turn, Prakashey et al. found an increased number of receding capillaries in the DR group compared with the DM group [34]. Moreover, some authors reported significant correlations between NVC parameters and DR severity, suggesting the method’s potential for grading disease progression [8,35,36]. Taken together, these results underscore the relevance of NVC as a valuable, noninvasive tool for assessing systemic and ocular microvascular impairment in DR. However, similar to glaucoma, NVC does not allow for the prediction of retinal changes in patients with scleroderma [37].

NVC has also been employed to investigate the relationship between NVC parameters and the occurrence of ocular manifestations, including uveitis, in systemic diseases with ocular involvement, such as Behçet’s syndrome and other connective tissue disorders. For example, Zontul et al. found that microhemorrhages and capillary crossings were associated with uveitis in Behçet’s syndrome patients, while no differences were observed for other NVC parameters [38].

The method has also been applied to patients with age-related macular degeneration (AMD). Studies have revealed distinct morphological alterations in the nailfold capillaries of individuals with AMD, including increased capillary ectasia, microhemorrhages, capillary tortuosity, neovascular formations, bizarre-shaped capillaries, and bushy capillaries [9].

Furthermore, NVC has found application in patients with idiopathic macular telangiectasia type 2 (MacTel2, also known as IMT). This neurodegenerative retinal disease is characterized by bilateral perifoveal telangiectatic vessels. A recent study compared proximal nailfold videocapillaroscopy (NVC) findings between 43 IMT (MacTel2) patients and 92 age- and gender-matched healthy controls. The results showed significantly higher rates of increased capillary tortuosity, microhemorrhages, and bizarre capillaries in IMT (MacTel2) patients. Furthermore, patients with microhemorrhages had higher superficial capillary plexus vascular density, while those with tortuous and bizarre capillaries showed lower subfoveal choroidal thickness [39]. The quantitative analysis by Gedik et al., reporting significant tortuosity and microhemorrhages in NVC (*p* < 0.001), suggests that MacTel2 may be associated with a systemic capillary remodeling process that parallels the telangiectatic changes observed in the macula [39].

In summary, nailfold videocapillaroscopy provides valuable insights into systemic microvascular changes associated with several ocular diseases. The consistent presence of capillary abnormalities such as hemorrhages, tortuosity, and avascular areas in glaucoma and central serous chorioretinopathy supports the concept of a shared vascular component in their pathogenesis. Although a few studies have reported no association, methodological differences and the presence of coexisting systemic vasculopathies may account for these discrepancies. Overall, NVC appears to be a promising, non-invasive tool that complements ocular imaging by revealing peripheral microvascular dysfunction potentially linked to eye disease severity and progression. Table 3 summarizes the main NVC findings associated with ocular disorders.

**Table 3:** Overview of NVC findings in patients with various ocular diseases.

Ocular Condition	Study Population	Main Findings from Nailfold Videocapillaroscopy (NVC)	Statistical Rigor/ Matching	Methodological Quality (NOS Score)	Ref.
glaucoma in patients with systemic sclerosis (SSc)	31 SSc patients including 7 with glaucoma age: 52.3 ± 11.6 (range 29–77 years)	no statistically significant association between NVC and glaucoma in SSc (*p* = 0.86)	Extremely small subgroup *n* = 7 for glaucoma; lack of statistical power; no age-adjustment Significant risk of selection bias due to systemic sclerosis-only cohort Pilot/Single-center study	7/9 (High)	[26]
primary open-angle glaucoma (POAG)	199 POAG patients 124 healthy controls age: 64.6 ± 8.9 vs. 62.5 ± 9.5 (*p* = 0.05)	capillary diameter: 14.5 ± 2.4 µm vs. 15.7 ± 1.6 µm (*p* = 0.036) increased presence of dilated capillaries (*p* = 0.002), avascular zones (*p* = 0.08) and hemorrhages (*p* < 0.001) in POAG	Multivariable Logistic Regression adjusted for age, sex, race, and comorbidity (borderline age-matching *p* = 0.05) The large cohort and robust adjustment protocols ensure high reliability Multisite study	9/9 (High)	[15]
primary open-angle glaucoma (POAG)	22 POAG patients 12 healthy controls age: 63.5 ± 9.4 vs. 69.9 ± 6.5 (*p* = 0.03)	lower nailfold capillary density in POAG (8.8 ± 1.0 vs. 9.8 ± 0.9 capillaries/mm; *p* = 0.009) lower nailfold capillary blood flow in POAG (19.9 ± 9.4 vs. 33.7 ± 9.8 pL/s; *p* = 0.0007) positive correlations between deep optic nerve head (ONH) and nailfold capillary blood flow (Pearson’s correlation coefficient *r* = 0.42, *p* = 0.02) and peripapillary and nailfold capillary density (*r* = 0.43, *p* = 0.03), and peripapillary and nailfold capillary blood flow (*r* = 0.49, *p* = 0.01)	GEE models adjusted for age, sex, race, and IOP. Accounted for inter-eye correlation. Controlled for systemic and ocular confounders Directly compared NVC with OCT-A parameters, demonstrating robust diagnostic validity Prospective study	9/9 (High)	[24]
primary open-angle glaucoma (POAG)	67 POAG patients 63 healthy controls age: 66 ± 8.6 vs. 67.2 ± 9.9 (*p* = 0.46)	reduced resting nailfold capillary blood flow in POAG (26.8 ± 17.6 pL/s vs. 50.1 ± 24.2 pL/s; <0.0001) independent of covariates such as blood pressure, pulse and IOP	Linear Mixed Models adjusted for age (*p* = 0.42), sex, race, and mean arterial pressure (MAP) Precise accounting for systemic factors and age-matching reliability. Prospective study	9/9 (High)	[18]
primary open-angle glaucoma (POAG)	83 POAG patients 40 healthy controls age: 70.2 ± 9.0 vs. 68.2 ± 8.1 (*p* = 0.23)	decreased capillary density in POAG (*p* = 0.002) increased presence of avascular zones in POAG (*p* = 0.01) no differences in dilated, crossed, and tortuous capillaries and hemorrhages Risk Factors: Avascular zones (OR = 1.24, *p* = 0.005), abnormal distribution (OR = 7.88, *p* = 0.001), and low density (OR = 0.63, *p* < 0.001). Severity Markers: Hemorrhages correlate with MD loss (β = −5.10, *p* = 0.015) and PSD (β = −4.37, *p* = 0.025).	Well-matched for age (*p* = 0.23) and sex. Logistic regression used to identify independent predictors of POAG. Strong evidence linking NVC abnormalities to functional visual field loss Multicenter Case–Control	8/9 (High)	[7]
primary open-angle glaucoma (POAG)	206 POAG patients 277 healthy controls age: 67.5 ± 10.6 vs. 63.2 ± 10.8 (*p* < 0.0001)	increased hemorrhages in POAG (*p* < 0.0001) increased dilated capillaries in POAG (*p* = 0.002) increased presence of avascular zones in POAG (*p* = 0.0005) presence of nailfold hemorrhages increases POAG risk over 6-fold (OR = 6.33; 95% CI: 2.14–18.70; *p* = 0.001). High prevalence in digits 4 and 5.	Multivariable Logistic Regression (adjusted for age (*p* = 0.26), sex, race). Masked observers during image analysis Identified nailfold hemorrhages as a potent independent biomarker for POAG Prospective, Case–control Study	8/9 (High)	[40]
Normal-tension glaucoma (NTG) Hypertension glaucoma (HTG) Primary angle closure glaucoma (PACG)	51 NTG patients 32 HTG patients 32 PACG patients 61 healthy controls age: 60.3 ± 15.6 POAG and 65.5 ± 11.5 PACG vs. 61.4 ± 14.8 Control (*p* = 0.671 and *p* = 0.148)	decreased capillary density, greater tortuosity, increased presence of dilated capillaries and avascular zones in POAG and PACG vs. control (for all *p* < 0.001) increased hemorrhages in POAG vs. control (*p* < 0.001) no differences in NTG vs. HTG NVC density correlates with peripapillary vessel density (r = 0.441, *p* < 0.001) and RNFL thickness. Reduced loop diameter in both POAG and PACG	GEE models (adjusting for inter-eye correlation, age, IOP); Age-matched: *p* = 0.671 (POAG) and *p* = 0.148 (PACG) Links nailfold morphology with retinal vessel density (strong cross-sectional evidence for systemic microvascular involvement) Prospective, Cross-sectional Study	8/9 (High)	[41]
Normal tension glaucoma (NTG)	80 NTG patients 50 age-matched controls, 58 young healthy controls age:65.09 ± 8.20 vs. 65.21 ± 5.31 vs. 30.06 ± 6.47 (age-matched)	more microvascular alterations in NTG (*p* = 0.0263) more frequent microbleedings in NTG (*p* = 0.0365), more frequent capillary enlargement in NTG (*p* = 0.0006) more frequent branching capillaries in NTG (*p* = 0.0221) less frequent meandering capillaries in NTG (*p* = 0.0082) high prevalence of avascular zones (27.5% vs. 0%, *p* < 0.001) and hemorrhages (46.3% vs. 14.3%, *p* = 0.001) correlation between NVC abnormalities and VF Mean Deviation.	Two control groups. Standardized Cutolo criteria Excellent age-matching (*p* = 0.39); Multidisciplinary assessment; Comparative analysis of specific NVC markers Robust evidence for peripheral microvascular involvement in the pathogenesis of NTG Prospective, Case–control Study	8/9 (High)	[16]
Normal tension glaucoma (NTG)	14 NTG eyes 15 healthy eyes age: 57.7 ± 12.6 vs. 54.5 ± 6.0 (*p* = 0.294)	significant reduction in capillary diameter (14.5 ± 2.4 μm vs. 15.7 μm; *p* = 0.036) and capillary length (*p* = 0.004) in NTG significant correlation between cold-induced blood flow reduction in ONH and NVC (r = 0.44, *p* = 0.01). Delayed recovery of capillary flow in NTG.	Functional cold provocation test; Excellent age-matching (*p* = 0.312); Innovative functional test with laser Doppler. Small sample size Robust physiological evidence for impaired systemic vasoreactivity in NTG patients Small-scale Mechanistic Study	8/9 (High)	[17]
pseudoexfoliative glaucoma (XFG): hypertensive (hXFG) normotensive (nXFG)	39 XFG patients (69 eyes) (hXFG: 54 eyes, nXFG: 15 eyes), 32 healthy controls age: 75.46 ± 7.63 XFG vs. 73.5 ± 5.21 (*p* = 0.748)	architectural derangement in XFG (*p* = 0.0332) increased capillary tortuosity in XFG (*p* = 0.0386) and nXFG (*p* = 0.0171) decreased number of capillaries in nXFG (*p* = 0.0297) increased frequency of microhemorrhages in nXFG (*p* = 0.0520) Microhemorrhages: 30.0% in nXFG vs. 6.25% in controls. Significant trend across the exfoliation spectrum	Precise phenotype differentiation; age-matched (*p* = 0.748 for XFG group); systemic blood pressure (BP) and sex matched Controlled for sex and comorbidities Strong evidence for systemic microvascular alterations Prospective, Case–control Study	8/9 (High)	[23]
exfoliation syndrome (XFS), exfoliation glaucoma (XFG) primary open-angle glaucoma (POAG)	56 XFS/XFG patients 87 POAG patients 75 healthy controls age: 72.2 ± 5.3 XFS/XFG and 64.8 ± 8.3 POAG vs. 64.8 ± 9.4 (*p* < 0.0001, *p* = 0.98)	more common nailfold hemorrhages, avascular zones and increased vascular tortuosity in XFS/XFG (*p* ≤ 0.0001) and POAG patients (*p* ≤ 0.01) vs. control more frequent avascular zones in XFS/XFG vs. POAG (*p* = 0.04) greater capillary tortuosity in XFS/XFG than in POAG patients (*p* = 0.005) OR = 11.4 (*p* < 0.001) for hemorrhages for XFS/XFG	GEE models adjusted for age (crucial since age *p* < 0.0001 for XFS/XFG) and excellent age-matching (*p* = 0.98) for the POAG vs. Control cohort Multisite Cross-sectional Study	9/9 (High)	[22]
exfoliation glaucoma (XFG), high-tension glaucoma (HTG) normal-tension glaucoma (NTG)	30 XFG patients 30 NTG patients 29 HTG patients 20 healthy controls age: 76.3 ± 6.9 XFG and 67.3 ± 11.6 NTG and 65.8 ± 9.7 HTG vs. 59.0 ± 12.8 (*p* < 0.001)	HTG, NTG, and XFG showed decreased peripheral blood flow at the nailfold of the fourth digit vs. control resting velocity: 0.23 mm/s in POAG and 0.20 mm/s in XFG vs. 0.42 mm/s in controls (approx. 50% deficit; *p* < 0.001) significant flow reduction: 30.6 pL/s in XFG, 40.1 pL/s in NTG, 47.5 pL/s in HTG vs. 70.9 pL/s in controls (univariate).	Linear Mixed Models adjusted for age disparity (*p* < 0.001). Results remained significant Linear Mixed Model adjustment for age and sex (confirmed that microvascular alterations remained independently significant) Multicenter Prospective Case–control Study	9/9 (High)	[25]
Central serous chorioretinopathy (CSC)	61 CSC patients 82 healthy controls age: 48.79 ± 11.15 vs. 49.38 ± 9.02 (*p* = 0.727)	decreased capillary density in CSC (<0.001) increased frequency of capillary ectasia, aneurysmal dilatation, microhemorrhages, avascular areas, tortuosity, neoformation, bizarre/bushy and meandering capillaries, extravasation (from <0.001 to <0.003)	Excellent demographic matching; age (*p* = 0.727) and gender (*p* = 0.933) were statistically comparable. Prospective, well-balanced groups Prospective, Case–control Study	7/9 (Moderate-to-High)	[30]
Central serous chorioretinopathy (CSC)	59 CSC patients 53 healthy controls age: 47.2 ± 9.4 vs. 46 ± 11.5 (*p* = 0.51)	significant overall capillary dilation in CSC (*p* = 0.004) and ramified capillaries (*p* = 0.000) and glomerular loops (*p* = 0.000) dilated apical part of capillaries, aneurysmal dilatations, bizarre loops, broken capillaries, megacapillaries, fresh hemorrhages—only in CSC	Well-matched for age (*p* = 0.51) and sex. Functional–structural correlation (OCT + Microperimetry) Robust evidence that peripheral microvascular abnormalities reflect the degree of retinal functional impairment in CSC Prospective Case–control Study	8/9 (High)	[31]
Central serous chorioretinopathy (CSC) acute (aCSC), recurrent (rCSC), chronic (cCSC), neovascular (nCSC)	43 aCSC patients 54 rCSC patients 44 cCSC patients 11 nCSC patients 41 healthy controls age: 46.30 ± 0.90 aCSC and 46.61 ± 0.94 rCSC and 47.50 ± 1.01 cCSC and 47.45 ± 2.08 nCSC vs. 47.12 ± 0.76 Control (*p* = 0.901)	abnormal NVC pattern in CSC (*p* = 0.000) ramified capillaries in CSC (*p* = 0.000) glomerular capillaries in CSC (*p* = 0.000), (more common in cCSC vs. aCSC (*p* = 0.000) and rCSC (*p* = 0.011); no difference vs. nCSC) dilated apical part of capillaries in CSC (*p* = 0.000), meandering capillaries in CSC (*p* = 0.035), (more frequent in aCSC and rCSC than in cCSC (*p* = 0.015)) aneurysmal dilatations are more common in nCSC than in aCSC (*p* = 0.009) and cCSC (*p* = 0.008)	Exceptional age-matching (*p* = 0.901) across 5 subgroups. Integration of advanced OCT analysis with NVC metrics provides a multi-layered validation of systemic vascular involvement in CSC Prospective Case–control Study	8/9 (High)	[32]
diabetic retinopathy (DR)	44 DR (+) 20 DR (−), T2DM age: 61.4 ± 7.4 vs. 56.6 ± 9.2 (*p* = 0.02)	more frequent capillary hemorrhage, ectasia, giant capillary, and neo-angiogenesis in DR group	ANOVA/T-test; Significant age difference noted (*p* = 0.02). Results require cautious interpretation regarding disease duration vs. aging (valuable for illustrating the progression of microvascular changes in T2DM) Cross-sectional, Case–control Study	7/9 (Moderate)	[33]
diabetic retinopathy (DR)	93 DR (+) 123 DR (−)/T2DM 101 Controls age: 60.89 ± 8.21 DR (+) and 58.92 ± 8.506 DR (−) vs. 59.41 ± 11.867 (*p* = 0.316)	increased: tortuosity (*p* = 0.002), bushy capillaries (*p* < 0.001), neoformation (*p* = 0.001) and capillary ectasia (*p* = 0.029) in DR tortuosity, bushy capillary, neoformation and capillary ectasia significantly higher in proliferative DR (*p* = 0.002, *p* < 0.001, *p* = 0.001 and *p* = 0.029)	Very large sample size. Perfect age-matching (*p* = 0.316) Detailed quantitative analysis (a significant correlation between NVC morphological markers and DR severity). Supports the diagnostic utility of NVC in monitoring systemic microvascular damage in DM Prospective, Case–control Study	8/9 (High)	[36]
diabetic retinopathy (DR)	100 T2DM (39 DR(+)/61 DR (−)) 100 controls Age stratification (25 per decade, 20–60 years)	increase in receding capillaries (*p* = 0.001); dilated capillaries (*p* = 0.022), neoangiogenesis (*p* = 0.003), meandering and tortuous capillaries (both *p* < 0.001), avascular zones (*p* = 0.007), and capillary density (*p* = 0.004) in T2DM increased receding capillaries in DR (*p* = 0.001) High correlation between capillary dropout and DR stages (*p* < 0.001)	Superior: Age stratification (25 participants per decade, 20–60 years). Perfect balance between groups (provides one of the strongest defenses against age-related confounding factors in NVC clinical application). Cross-sectional Comparative Study	8/9 (High)	[34]
diabetic retinopathy (DR)	26 DR (+) (22 NPDR/4PDR) 36 T2DM/DR (−) age: Not reported (NR) focus on disease duration (16.6 vs. 7.8 years)	more frequent branched capillaries and tortuous capillaries in DR (*p* = 0.01 for both) more frequent microhemorrhage (*p* = 0.053) and precapillary edema (*p* = 0.024) in DR increased capillary width in DR (*p* = 0.001) dilated apical capillaries more frequent in proliferative DR (*p* = 0.004) significant correlation between capillary density and DR severity (*p* < 0.001) branched capillaries and abnormal capillary width were significantly associated with DR (OR 8.349, *p* = 0.004; OR 1.353, *p* = 0.001)	Age matching not reported (NR). Potential age-related bias due to disease duration disparity Despite reporting limitations, the study provides significant correlation data between NVC and ETDRS-graded retinopathy Case–control Study	7/9 (High)	[42]
Age-related macular degeneration (AMD)	53 patients 91 controls age: 70.45 ± 7.49 vs. 68.75 ± 4.33 (*p* = 0.085)	increased capillary ectasia (*p* = 0.017), micro-hemorrhages, tortuosity, neo-formation, bizarre and bushy capillaries in AMD (<0.001 for all) no significant differences for capillary aneurysm in AMD no significant differences between dry and wet types of AMD	Groups well-matched for age and sex (*p* = 0.113). Selection bias minimized. Confirms that systemic microvascular alterations in AMD are independent of aging. Prospective, Case–control Study	8/9 (High)	[9]
Uveitis	107 uveitis patients 130 healthy controls age: 36.9 ± 12.5 vs. 36.2 ± 12.2 (*p* = 0.69)	higher tortuosity ratings and reduced capillary density in uveitis (*p* < 0.001 for both); dilated capillary loops, avascular zone and hemorrhages were more frequent in uveitis (*p* < 0.001 for all) increase in capillary density associated with disease activity (OR 1.73, *p* = 0.013) haemorrhagies with posterior and panuveitis (OR = 5.83, *p* < 0.001)	Age-matched (*p* = 0.69). Advanced multivariate logistic regression (OR analysis). Young cohort (avg. 36 years) High methodological rigor ensures that NVC abnormalities reflect uveitis-related pathology rather than aging. Cross-sectional Observational Study	8/9 (High)	[43]
Uveitis	119 patients 25 pediatric controls age: 13.7 ± 3 vs. 9.1 ± 4 (*p* < 0.001)	dilated and ramified capillaries more frequent in uveitis (*p* = 0.04 and *p* = 0.02), no significant differences for other NFC parameters a longer duration of uveitis in patients with a capillary density <7/mm higher capillary density in patients with papillitis compared to those without papillitis (*p* = 0.04); lower capillary density and more microhemorrhages in ANA-positive patients compared to ANA-negative patients. (*p* = 0.02 and *p* = 0.04)	Regression analysis adjusted for age and sex. Pediatric cohort eliminates aging as a confounder. High methodological rigor in assessing systemic microvascular involvement in pediatric uveitis. Cross-sectional study	8/9 (High)	[44]
Uveitis	25 patients 21 pediatric controls age: 11.24 ± 3.03 vs. 9.9 ± 4.17 (*p* = 0.22)	lower capillary density in uveitis (*p* = 0.002) no significant differences for other parameters	Age and gender-matched (*p* = 0.22 for age). Mean capillary density showed no significant correlation with age (*p* = 0.937) Pediatric cohort eliminates aging as a confounder. Case–control study	8/9 (High)	[45]
Uveitis (Behçet’s Syndrome (BS)	32 BS patients uveitis (+) 29 BS patients uveitis (−) 29 health controls age: 46 46.2 ± 10.7 and 40.6 ± 11.8 vs. 40.3 ± 10.9 (*p* = 0.072)	significant increase in crossing capillaries (median 2.0 vs. 1.2; *p* < 0.001), microhemorrhages (9 vs. 1; *p* = 0.028)	Groups statistically matched for age (*p* = 0.072) and gender (*p* = 0.948). Case–control study	8/9 (High)	[38]
Retinal vein occlusion	30 patients 30 health controls age: 69.5 vs. 66.5 (*p* = 0.61)	higher tortuosity (*p* = 0.003) and reduced capillary density (*p* < 0.001) in RVO patients, dilated capillary and avascular zones more frequent in RVO	Excellent matching for age, sex, and comorbidities (*p* > 0.05). Precise quantitative metrics for vessel diameters and RVD correlations. Cross-sectional study	8/9 (High)	[46]
Idiopathic macular telangiectasia type 2 (MacTel2, IMT)	43 MacTel2 (IMT) patients 92 healthy controls age: 59.76 ± 5.73 vs. 58.23 ± 4.96 (*p* = 0.69)	increased capillary tortuosity in MacTel2 (IMT) (*p* < 0.001) microhemorrhages—only in MacTel2 (IMT) (*p* < 0.001) bizarre capillaries in MacTel2 (IMT) (*p* < 0.001)	Excellent age-matching (*p* = 0.69). Mean age ~60 in both groups Prospective, Case–control Study	8/9 (High)	[39]

Abbreviations used in Table 3: NOS—Newcastle–Ottawa Scale (scores of 7–9 indicate high quality, 4–6 moderate quality, and <4 low quality); SSc—Systemic sclerosis; POAG—Primary Open-Angle-Closure Glaucoma; GEE—Generalized Estimating Equations; IOP—Intraocular Pressure; NTG—Normal-Tension Glaucoma; HTG—High-Tension Glaucoma; PACG—Primary Angle-Closure Glaucoma; XFG/XFS—Pseudoexfoliation Glaucoma/Pseudoexfoliation Syndrome (hXFG—high tension XFG, nXFG—normal tension XFG); CSC (aCSC, rCSC, cCSC, nCSC)—Central Serous Chorioretinopathy (acute, recurrent, chronic, neovascular types); DR—Diabetic Retinopathy (NPDR—non-proliferative DR; PDR—proliferative DR); T2DM—Diabetes Mellitus type 2; OR—Odds Ratio, MD—Mean Deviation; PSD—Pattern Standard Deviation; RNFL—Retinal Nerve Fiber Layer; NR—Not Reported.

## 5. Limitations of Reviewed Literature

A critical strength of the reviewed evidence is the prevalence of prospective, case–control designs (e.g., Latalska et al., Uyar et al., Gedik et al.), which allow for a more robust causal inference than retrospective reports [32,36,39]. In these studies, the ‘meaningfulness’ of comparison groups was ensured through strict age- and sex-matching, often reaching near-perfect demographic balance (e.g., *p* = 0.69 in Chen et al. and *p* = 0.419 in Gedik et al.) [39,43]. Furthermore, recent studies of young populations (e.g., uveitis patients, mean age 36) have definitively dissociated NVC abnormalities from age-related effects on vessel condition [37]. While long-term prospective data remain limited for certain conditions, such as MacTel2, the current cross-sectional evidence—supported by high Odds Ratios (up to 5.8 for microhemorrhages)—provides a solid foundation for the clinical utility of NVC as a systemic biomarker in ophthalmology.

Despite these strengths, a critical assessment of the included studies reveals significant heterogeneity in study design and quality, which requires caution in interpreting the results.

Firstly, there is a substantial disproportion in sample sizes, ranging from small pilot studies (*n* < 20) to larger cohorts (*n* > 200). Several studies failed to adequately control for key confounding factors, particularly age and systemic blood pressure, which are known to independently alter NVC parameters. Apart from age, cardiovascular comorbidities (such as hypertension, atherosclerosis, or heart failure) are known to independently alter NVC parameters, leading to structural changes, such as reduced capillary density or increased tortuosity [5,21]. Since these conditions are highly prevalent in the elderly population—who also constitute the majority of patients with glaucoma or AMD—distinguishing disease-specific ocular microangiopathy from generalized cardiovascular aging remains a major challenge.

Secondly, a methodological concern arises regarding the statistical handling of data. Many studies explored correlations between numerous NVC parameters (e.g., density, loop diameter, hemorrhages) and multiple OCT-A metrics. This approach increases the risk of Type I errors (false positives) due to the multiple comparisons problem. Few studies applied strict corrections (e.g., Bonferroni adjustment) to account for this. Consequently, reported associations should be viewed as exploratory and hypothesis-generating rather than definitive evidence of a causal link.

The clinical validity of NVC studies is determined by methodological quality. Although foundational studies have contributed valuable data, many are limited by inconsistent demographic matching, with *p*-values for age often near 0.05. To progress beyond these preliminary exploratory phases, future research should adopt rigorous standards. Specifically, robust protocols should be employed to ensure *p* > 0.05 for all baseline characteristics and multivariate models to distinguish true eye-specific microangiopathy from confounding systemic vascular aging effects. A prime example of such methodological rigor is the deliberate stratification of age groups or the inclusion of pediatric cohorts (e.g., Kouwenberg et al. [44], Abdelrahman et al. [45]), which allows for a definitive dissociation of disease-specific NVC patterns from age-related capillary remodeling. Furthermore, incorporating longitudinal data would clarify whether these systemic microvascular biomarkers can predict ocular disease progression over time.

Finally, the inherent limitations of NVC itself must be acknowledged. The method is susceptible to external factors such as room temperature, local trauma (e.g., manicures), and skin transparency, which can affect image quality and interpretability.

## 6. Conclusions and Future Perspectives

The presented systematic review confirms that nailfold videocapillaroscopy (NVC) extends beyond its traditional role in rheumatology and is becoming a valuable, though still underappreciated, tool in ophthalmic diagnostics. The assembled evidence indicates a clear association between systemic microcirculatory changes observed in NVC and pathologies of the retina, choroid, and optic nerve.

The consistent demonstration of abnormal capillary patterns in patients with glaucoma (especially normal-tension glaucoma), central serous chorioretinopathy (CSC), and diabetic retinopathy (DR) strongly supports the hypothesis of a common, systemic component of endothelial dysfunction and vascular dysregulation underlying these seemingly distinct ocular diseases.

The identification of these links opens new, fascinating clinical and research perspectives:

NVC as a risk stratification tool: The most promising application appears to be the use of NVC as a non-invasive screening tool. In diabetic retinopathy, studies have shown not only differences in NVC patterns between diabetic patients with and without retinopathy, but also a correlation with the severity of DR. NVC may thus aid in the early identification of diabetic patients at higher risk who require more intensive ophthalmic monitoring, potentially even before clinically evident retinal changes occur.

A key perspective is the synergy between NVC and modern ocular imaging, particularly OCT-A. Integrating these methods is essential to clarify how systemic endothelial vulnerability leads to ocular pathology. For example, studies such as Latalska et al. in CSC patients and Gedik et al. in MacTel 2 (IMT) patients highlight the clinical value of this dual-imaging approach [32,39]. The former found significant correlations between NVC and OCT-A parameters, including foveal avascular zone (FAZ) dimensions and retinal plexus density, and also linked tortuous capillaries to choroidal pachyvessel diameter [30]. Similarly, Gedik et al. reported that in MacTel 2 (IMT) patients, NVC microhemorrhages correlated with superficial capillary plexus vascular density, while tortuous and bizarre capillaries correlated with lower subfoveal choroidal thickness [39]. These findings suggest that systemic microvascular remodeling, though governed by mechanisms different from those of retinal circulation, parallels the severity of localized ocular vascular disturbances.

This multitude of reported associations, linking specific NVC morphologies with precise OCT-A metrics, is intriguing and strongly supports the hypothesis of a shared pathophysiology. However, these results must be approached with significant methodological caution. Analyzing such a large number of NVC variables against numerous OCT-A parameters (across multiple sectors and for both eyes) carries a high risk of the multiple comparisons problem and yielding false-positive results (a Type I statistical error). To minimize this risk, rigorous inclusion criteria and well-defined anatomical sectors were employed in high-quality studies included in this review to strengthen the validity of the reported correlations despite the inherent statistical complexity (e.g., Latalska et al. and Gedik et al.) [32,39].

Therefore, while these preliminary correlations are invaluable for hypothesis generation, future research must focus on their validation in larger cohorts, with the mandatory application of appropriate statistical corrections (e.g., Bonferroni or Benjamini–Hochberg).

Standardization and Artificial Intelligence (AI): The complexity of morphological patterns described in the literature—such as spiraled, meandering, bushy, or bizarre capillaries—poses a challenge for objective interpretation. However, this is an ideal field for the application of Artificial Intelligence (AI) algorithms. Automated analysis of NVC images using AI could offer objective quantification of changes and identify subtle risk patterns that the observer may currently miss.

Finally, the use of NVC in ‘Smart Healthcare’ represents a significant future direction. As discussed in recent reviews on ‘Artificial Intelligence in Smart Healthcare,’ combining capillary imaging with deep learning could allow for home-based monitoring of patients with chronic systemic microvascular and ocular diseases [47]. However, confirming this potential requires advancing beyond small cross-sectional observations. Large-scale, longitudinal population studies are necessary to minimize Type I errors and to confirm the true predictive value of NVC for eye disease progression.

In conclusion, NVC has the potential to become a valuable diagnostic adjunct for the ophthalmologist, offering a unique “window” into the patient’s systemic microcirculatory status. However, to fully translate these promising research findings into daily clinical practice, further standardization of methodology and validation studies on large patient cohorts are necessary.

## Figures and Tables

**Table 1:** Comparison of nailfold videocapillaroscopy and nailfold capillaroscopy.

Feature	Nailfold Capillaroscopy (NFC)	Nailfold Videocapillaroscopy (NVC)
Magnification	Typically ×10–×40	Usually ×200–×600
Equipment	Handheld microscope, dermatoscope, or ophthalmoscope	Video-capillaroscope with optical probe and computer-assisted analysis software
Image capture	Visual observation, sometimes with simple photography	Digital image acquisition with storage, measurement, and quantitative analysis
Assessment	Mainly qualitative (general morphology, gross abnormalities)	Quantitative and qualitative (density, dimensions, tortuosity, flow patterns)
Reproducibility	Lower, observer-dependent	High, due to standardized image capture and measurement software
Data analysis	Manual, subjective	Automated or semi-automated, objective and repeatable

**Table 2:** Conceptual parallel of systemic NVC morphologies and their potential ophthalmic counterparts.

Parameter	Pathophysiological Meaning	Possible Ocular Significance
Capillary density	Reflects the number of functioning capillary loops per millimeter; decreased density indicates capillary loss or reduced perfusion.	Suggests systemic microvascular rarefaction; correlated with reduced retinal, choroidal, and optic nerve blood flow
Capillary morphology	Structural irregularities (tortuosity, bushy or dilated loops) indicate endothelial dysfunction and vascular dysregulation.	May parallel morphological changes in retinal or choroidal vessels observed in microangiopathies and ischemic ocular diseases.
Giant/dilated capillaries	Represent compensatory vasodilation due to hypoxia or inflammatory activation.	Analogous to compensatory vascular dilation in choroidal ischemia or early stages of diabetic retinopathy.
Microhemorrhages	Result from fragility or rupture of capillary walls, reflecting endothelial damage.	May correspond to microvascular leakage and hemorrhages seen in retinal vein occlusion or diabetic microangiopathy.
Avascular areas	Indicate loss of capillary loops and local ischemia.	Mirror focal hypoperfusion or ischemic zones in retinal and choroidal circulation.
Neoangiogenesis	Reflects chronic hypoxia-induced formation of new, often disorganized vessels.	Comparable to angiogenic processes in proliferative retinopathies.

## Data Availability

The data presented in this study are within the article.
